# Tracing intensive fish and meat consumption using Zn isotope ratios: evidence from a historical Breton population (Rennes, France)

**DOI:** 10.1038/s41598-018-23249-x

**Published:** 2018-03-22

**Authors:** Klervia Jaouen, Rozenn Colleter, Anita Pietrzak, Marie-Laure Pons, Benoît Clavel, Norbert Telmon, Éric Crubézy, Jean-Jacques Hublin, Michael P. Richards

**Affiliations:** 1 Department of Human Evolution, Max Planck Institute for Evolutionary Anthropology, Leipzig, Germany; 2 INRAP, Cesson-Sévigné, France; 3 AMIS, UMR 5288, Université Paul Sabatier, Toulouse, France; 4 Department of Earth Sciences, University of Cambridge, Cambridge, United Kingdom; 5 CNRS/MNHN/Sorbonne Universités, UMR 7209 Paris, France; 6 Department of Forensic Medicine, CHU Toulouse Rangueil, Toulouse, France; 7 Department of Archaeology, Simon Fraser University, Vancouver, Canada; 8 Department of Geosciences, University of Tübingen, Tübingen, Germany

## Abstract

Here we report Sr and Zn isotope ratios of teeth of medieval to early modern Breton people a population whose diet is known from historical, archeological and collagen isotope data. Most of the population, buried in the Dominican convent of Rennes, France, consists of parliamentary nobles, wealthy commoners and ecclesiastics, who had a diet rich in animal products. Our aim is to assess how the Zn isotope ratios of their teeth compare to those of other French historical populations previously studied, which were characterized by cereal-based diets, and those of modern French individuals, who daily eat animal products. We describe a clear offset (∼0.35‰) between local and non-local human individuals in Zn isotope ratios. The δ^66^Zn_tooth_ values of local individuals overlap that of modern French people, and are lower than those of local carnivores. Non-local δ^66^Zn values are similar to those of historical individuals analyzed previously. We conclude the lower Zn isotope ratios of local humans relative to the associated fauna can be explained by the consumption of carnivorous fish and pork, in agreement with historical, zooarchaeological and collagen (C, N, S) isotope data. Zn isotopes could therefore be a tracer of fish and/or substantial meat consumption in ancient populations.

## Introduction

The origin of Zn isotopic variability in human tissues remained unknown until Van Heghe *et al*. (2012)^1^, reported the strong impact of meat and fish consumption on blood Zn isotope ratios (^66^Zn/^64^Zn expressed as δ^66^Zn values), a preliminary conclusion quickly confirmed by Costas-Rodriguez *et al*. (2014)^2^. A parallel study on African food webs did not quantify the exact relationship between diet and bone Zn isotope ratios^3^, however by focusing on a much smaller geographical area, the sensitivity of Zn isotopes to diet was demonstrated^4^: Zn isotope ratios of bones and teeth clearly differ between carnivores and herbivores, with carnivores exhibiting the lowest ratios. The dependence of Zn isotope ratios on trophic level has also been confirmed in a marine ecosystem^5^.

The isotopic composition of Zn in animal tissues is controlled by two dietary factors: the isotopic fractionation that occurs during intestinal absorption and the Zn isotope ratios of the food products. Dietary Zn mainly comes from animal products, notably because Zn – and preferentially its lighter isotopes - from plants tends to precipitate with the phytates in the gastro intestinal tract^6^. This precipitation is likely to trigger isotopic fractionation inducing the preferential absorption of heavy Zn isotopes. Additionally, plant products usually have the most elevated δ^66^Zn values^2^. As a consequence, herbivore tissues exhibit higher Zn isotope ratios compared to carnivore or omnivore tissues^3–5^. Muscles are ^66^Zn depleted relative to the average isotopic composition of the body and no isotope fractionation of Zn is expected during meat consumption^3^. Carnivores therefore have lower δ^66^Zn values than their prey: the higher the trophic level of an animal is, the lower are the Zn isotope ratios of its body tissues^5^.

Zn isotope ratios of dental enamel from populations from different locations and historical periods were recently compared^7^. The study highlighted a very surprising trend: the δ^66^Zn dental values of preindustrial populations were much higher than those of modern individuals. Two explanations were then hypothesized to explain such a pattern. The observed trend could be due to:

1. an increase in fish and meat consumption in the 20^th^ century^8^. As mentioned above, elevated Zn isotope ratios are expected in tissues of individuals with plant-based diets^1–4^. Conversely, consumption of high trophic level food products, such as carnivorous fish (e.g. tuna, salmon, cod, pike) is expected to generate low Zn isotope ratios of mammal tissues.
2. the release of anthropogenic Zn in modern environments by industries and/or the use of manuring products. The anthropogenic Zn can indeed exhibit low Zn isotope ratios^9,10^ and enter modern food webs^9^.

**Figure 1:**
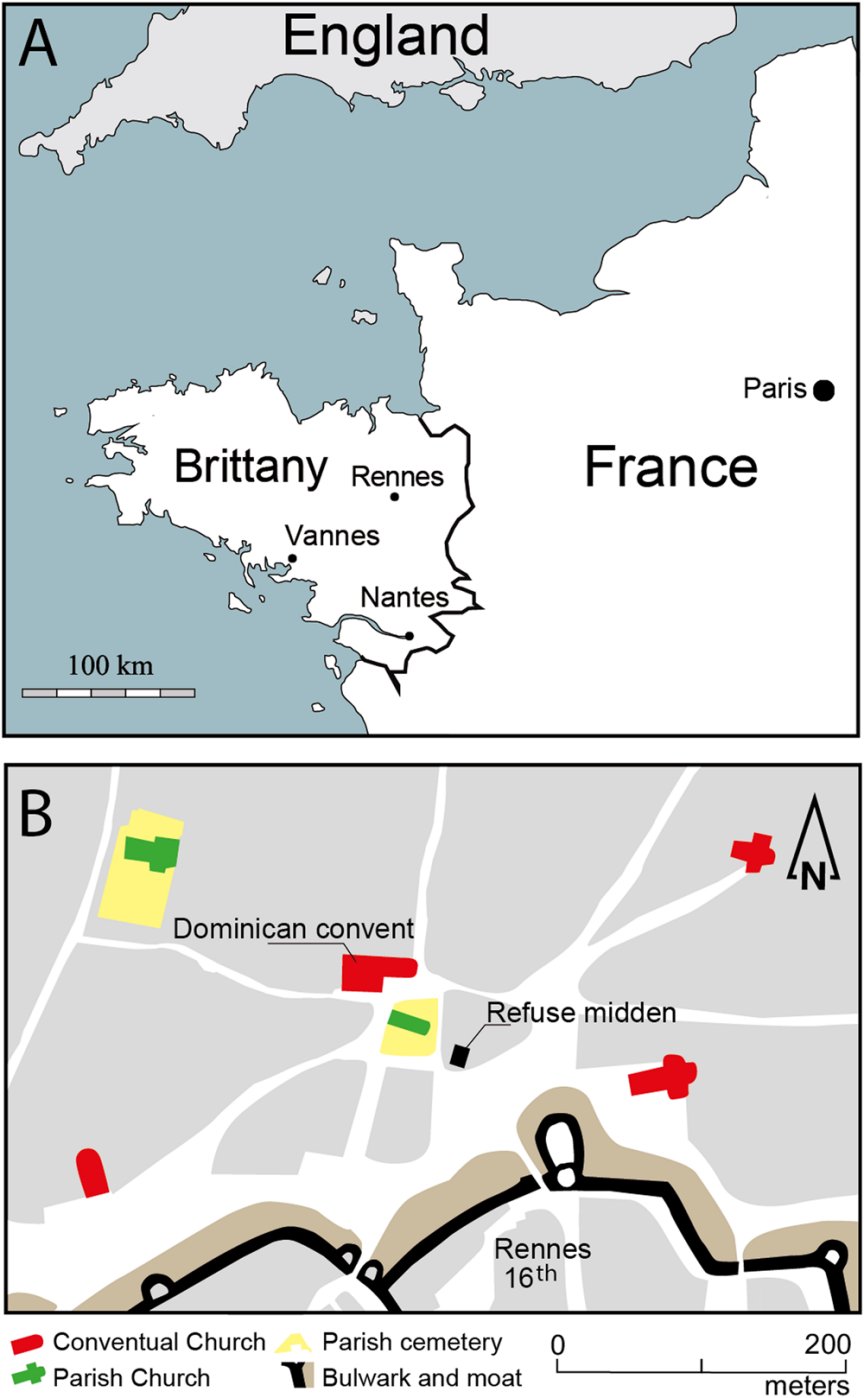
Location of Rennes and its Dominican convent. (**A**) Historical border of Brittany in the 15^th^ century (blank map fron Daniel Dalet, http://www.histgeo.ac-aix-marseille.fr). (**B**) Location of the convent and the refuse dump outside of the walls in the 16^th^ century (after the city map drawn by Hévin in 1685, image free of rights, http://www.wiki-rennes.fr/Fichier:Plan_Hevin.jpg). Both maps were created using the software Adobe Illustrator CS6.

In order to decide between these two hypotheses, we analyzed a preindustrial population (13^th^ to 18^th^ century) characterized by diets with significant meat and fish consumption^11^. This allows us to test if historic human population living before the release of anthropogenic Zn into modern environments exhibit δ^66^Zn values closer to other preindustrial populations characterized by a cereal-based diet, or are more like modern individuals who also had diets with intensive meat and fish consumption. We studied the wealthy/elite late medieval to early modern population of the former Dominican convent of Rennes (Brittany, France, Fig. 1), for which we already reconstructed the diet using C, N and S isotopes^11^. This population includes ecclesiastics, nobles and commoners who had to obey fasting rules and therefore ate fish instead of meat for one third of the year^12^. The medieval and early modern diets of the aristocracy also included a very important amount of meat on non-fasting days. The C, N and S isotope ratios determined from the bones and teeth on the individuals buried in Rennes’s Dominican convent as well as the zooarchaeological study performed on this site and the nearby refuse midden, indicate a substantial consumption of animal products: terrestrial herbivores and omnivores, including suckling pigs, eels and marine fish^11^.

Since the environmental context can impact local Zn isotope ratios^4,9,13^, the geographical origin of the individuals was assessed using Sr isotope ratios in human dental enamel as well as previously published S isotope data obtained on tooth collagen (Supplementary Information 1). Potential impacts of marine food consumption on S and Sr isotope ratios are assessed using previously published δ^13^C values^11^ by evaluating the absence or presence of a correlation between these isotope ratios.

In this paper, we present stable Zn and radiogenic Sr isotope data from the analysis of dental enamel of 54 individuals from the Dominican convent of Rennes. The associated fauna (6 terrestrial animals) was also analyzed to better interpret the diet of this population and compared the isotopic data of C, N and S previously conducted on the collagen from the same teeth.

## Results

A socio-economic group was attributed to the different individuals depending on their burial location^11^. The majority are elite and wealthy individuals who are buried in the church and its chapels (group *Privileged*, A), but three subgroups can be defined within this group. The first subgroup (A’) includes the aristocrats identified by discriminatory funeral practices (embalming and/or lead coffins)^11,14^. A second subgroup is identified as slightly less favored as the others (B’) and includes the individuals buried in the nave of the church. The rest of the individuals buried in the church choir and the chapels corresponds to the last subgroup (A-A’). The ‘non-privileged’ individuals are buried outside the walls of the convent in the cloister garden and in the immediate exteriors of the convent (group *Non-privileged*, B-B’). In addition, the individuals from the chapter house are likely to be Dominican ecclesiastics (group *Ecclesiastics*, C). Finally, men with blade injuries found in mass graves out of the convent’s walls were probably soldiers (group *Soldiers*, D). The distribution of the individuals sampled among socio-economic group, phase, age at death and sex are given in the Table 1. Results for Sr and Zn isotope ratios measured in animal and human bones and teeth are given for each individual in the Supplementary Information Tables S2 and S3 and summarized in the Table 2. Isotope compositions of external standards are also described in the Supplementary information (Table S3) and are in agreement with expected values.

**Table 1:** Distribution of the individuals sampled among socio-economic group, phase, age of death and sex.

	I	J	F	M	PM	All
**Non-privileged (B-B’)**						
Phase 1	0	1	0	3	0	4
Phase 2	0	0	1	5	0	6
Phase 3	0	0	0	0	0	0
All non-privileged	0	1	1	8	0	10
**Privileged (A-A’, A’, B’)**						
Phase 1	0	0	0	0	0	0
Phase 2	1 (A’)	0	1 (A’)	0	0	2 (A’)
Phase 3	3	3	8	15	3	32
A'	0	1	3	2	0	6
A-A'	1	1	3	9	3	17
B'	2	1	2	4	0	9
All privileged	4	3	9	15	3	34
**Ecclesiastics (C)**						
Phase 1	0	0	0	0	0	0
Phase 2	1	0	0	1	1	3
Phase 3	0	0	0	3	1	4
All ecclesiastics	1	0	0	4	2	7
**Soldiers (D)**						
Phase 1	0	0	0	0	0	0
Phase 2	0	0	0	3	0	3
Phase 3	0	0	0	0	0	0
All soldiers	0	0	0	3	0	3

F: female, M: male, PM: probable male, I: indeterminate, J: juvenile.

**Table 2:** Sr, Zn and N isotope ratios of human and animal teeth depending on the socio-economic group of the individuals buried in the Dominican convent of Rennes, Brittany.

	δ^66^Zn	2 SD	n	^87^Sr/^86^Sr	2 SD	n	δ^15^N	2 SD	n
**Animals**									
Carnivore (cat)	0.66	—	1	0.7127	—	1	11.2	—	1
Omnivores (pig and dog)	0.58	0.14	2	0.7116	0.0002	2	11.9	2.4	2
Herbivores (cow and sheep)	0.96	0.24	3	0.7136	0.0022	3	8.7	1.8	3
**Probable locals**									
Non-Privileged (B-B’)	0.34	0.26	5	0.7119	0.0018	5	12.5	1.8	5
Ecclesiastics (C)	0.35	0.28	8	0.7115	0.0016	8	13.2	1.4	8
Privileged (A, B’)	0.31	0.30	30	0.7116	0.0016	30	13.2	2.2	25
Soldiers (D)	0.51	—	1	0.7109	—	1	13.1	—	1
All	0.33	0.28	45	0.7116	0.0016	45	13.2	2.0	39
**Non-locals**									
Non-Privileged (B-B’)	0.69	0.26	4	0.7093	0.0010	4	12.0	1.0	3
Ecclesiastics (C)	0.72	—	1	0.7096	—	1	11.5	—	1
Privileged (A, B’)	0.63	0.82	3	0.7094	0.0004	3	13.0	1.8	3
Soldiers (D)	0.70	—	1	0.7091	—	1	10.2	—	1
All	0.68	0.44	9	0.7093	0.0008	9	12.1	2.2	8

### Sr isotope analyses

**Figure 2:**
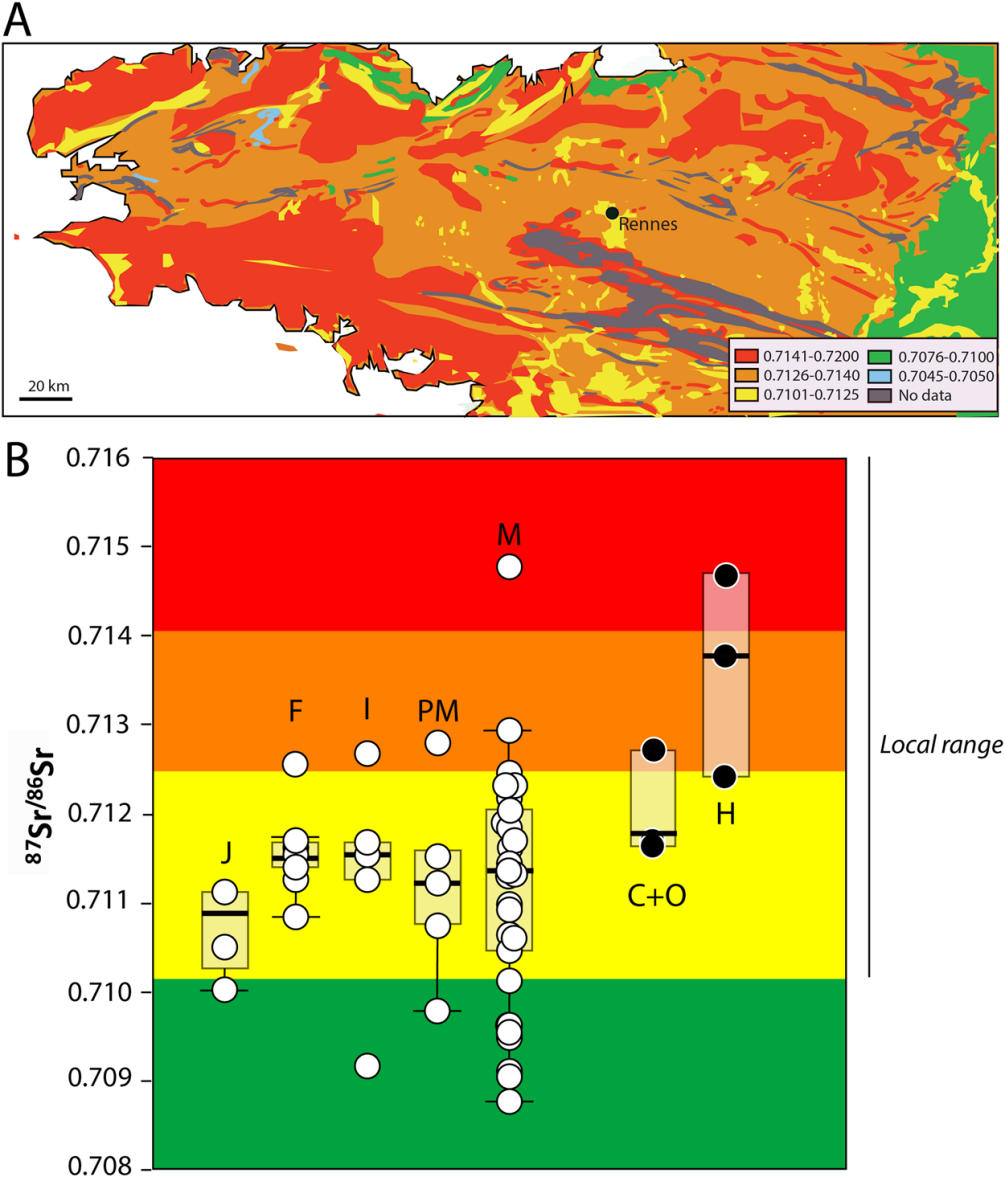
Sr isotope ratios in Brittany (according to IRHUM database) and in dental enamel of Rennes individuals. J: juveniles. F: female. I: Indeterminate. PM: probable male. M: male. C+O=carnivorous and omnivorous animals. H: herbivores. The error bars are smaller than the symbol size. The map of Brittany and associated Sr isotope ratios was created using Adobe Illustrator CS6 based on the information available on the IRHUM database website^16^ (http://80.69.77.150/, blank map fron Daniel Dalet, http://www.histgeo.ac-aix-marseille.fr).

The Sr isotope ratios of terrestrial mammal teeth (n = 6) range from 0.7115 and 0.7146. Terrestrial domestic animals (pig, cat, dog, cows and sheep) being raised in the city or in the nearby country^15^, have local bioavailable ^87^Sr/^86^Sr values. This range also fits with the local values expected from the IRHUM database^16^ (Isotopic Reconstruction of Human Migration), ranging from 0.710 to 0.716 (Fig. 2A). The IRHUM database documents a really limited sea spray effect on the coasts of Brittany (Fig. 2B). The individuals exhibiting, lower, “non-local” tooth values are supposed to have spent the end of their childhood or the beginning of their adolescence in a sedimentary or volcanic region. Seventeen percent of the individuals analyzed are exhibiting non-local ^87^Sr/^86^Sr (^87^Sr/^86^Sr < 0.710 based on the subdivisions from the IRHUM database reproduced in Fig. 2), and are therefore likely to have spent their childhood outside of Brittany (9/54 teeth). By combining S isotopes that provide information on the relative distance to the coast – or more exactly, the existence of a marine influence on local values^17^ - to Sr isotopes, tracers of the local geology, 6 categories of geographical origins were defined/can be defined (Table 3). Two individuals exhibit inland S isotope values (NC-non-coastal) - therefore are not compatible with the observed Breton range- but ^87^Sr/^86^Sr > 0.710 (R-radiogenic) –compatible with the local range (R_NC). One individual is characterized by a coastal (C, δ^34^S > 11‰)^11,17^ but non-local Sr (NR-non-radiogenic, ^87^Sr/^86^Sr < 0.710) isotope signature (NR_C). Four individuals have both non-local S and Sr isotope values (inland δ^34^S value and ^87^Sr/^86^Sr < 0.710. NR_NC). Finally, for the remaining four individuals with non-local Sr isotope ratios, the S isotope signature of their teeth was not available (NR_X). As previously observed with S isotopes, all women and nobles tested (group 2 A’ and 3 A’, Table S1) fall into the “probable local” group (n_women_ = 10, n_nobles_ = 8). All soldiers showed non-local S isotope values in teeth or bones (n_soldiers_ = 3) but only one of the three teeth analyzed exhibit a ^87^Sr/^86^Sr value incompatible with the local range. One ecclesiastic from the second phase, already spotted as non-local with its δ^34^S value^11^, is also characterized by a non-local ^87^Sr/^86^Sr, whereas other ecclesiastics exhibit values compatible with the local range (6/7 ecclesiastics). The other non-local individuals mostly consist of people from the less-privileged groups (1B, 2B, and 3B’) (4 non-privileged for 9 non-local individuals).

**Tablee 3:** Sr, Zn, C and N isotope compositions of human teeth depending on the geographical origin of the individuals buried in the Dominican convent of Rennes, Brittany.

	δ^66^Zn	2 SD	n
	Average		
NR_C	0.81	—	1
NR_NC	0.65	0.18	4
NR_X	0.68	0.84	4
R_C	0.33	0.28	21
R_NC	0.33	0.02	2
R_X	0.32	0.30	22
	^**87**^ **Sr/** ^**86**^ **Sr**		
	**Average**	**2 SD**	**n**
NR_C	0.7091	—	1
NR_NC	0.7093	0.0011	4
NR_X	0.7096	0.0008	4
R_C	0.7116	0.0011	21
R_NC	0.7121	0.0002	2
R_X	0.7115	0.0016	22
	** δ** ^**15**^ **N**		
	**Average**	**2 SD**	**n**
NR_C	12.4	—	1
NR_NC	11.8	2.4	4
NR_X	12.8	2.2	3
R_C	13.3	2.0	21
R_NC	12.2	1.8	2
R_X	13.1	2.0	16
	**δ** ^**13**^ **C**		
	**Average**	**2 SD**	**n**
NR_C	−19.5	—	1
NR_NC	−19.2	0.5	5
NR_X	−18.9	0.6	2
R_C	−19.3	0.6	21
R_NC	−18.7	0.1	2
R_X	−19.2	0.9	16

Results are grouped according to the geographical origin of the humans, assessed from the S and Sr isotope compositions of their teeth. NR_C: coastal δ^34^S and ^87^Sr/^86^Sr < 0.71; NR_NC: non-coastal δ^34^S and ^87^Sr/^86^Sr < 0.71; NR_X: unknown δ^34^S value and ^87^Sr/^86^Sr < 0.71; R_C: coastal δ^34^S and ^87^Sr/^86^Sr > 0.71; R_NC: non-coastal δ^34^S and ^87^Sr/^86^Sr > 0.71; NR_X: unknown δ^34^S value and ^87^Sr/^86^Sr > 0.71. C stands for coastal, R for radiogenic, N for non-.

### Zn isotope analyses

**Figure 3:**
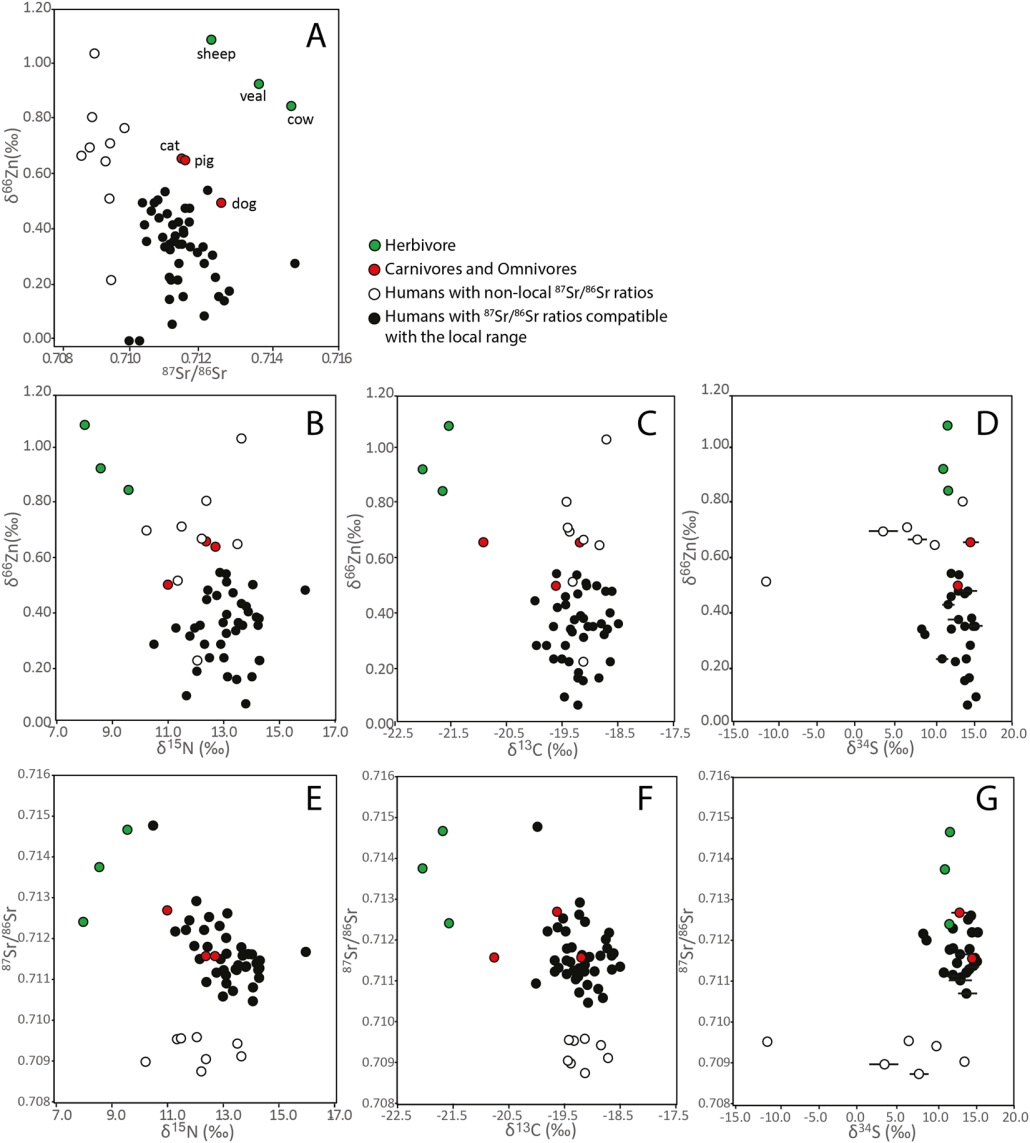
Isotope ratios in teeth of humans and animals buried in Rennes, Brittany. C, N and S isotope ratios were measured in dentine collagen and published in a previous work^11^. Zn and Sr isotope ratios were measured in dental enamel. Error bars are represented when they exceed the symbol size.

As previously observed, Zn isotope ratios of dental enamel do not correlate with Zn concentrations^7,18^. This shows the absence of a mixing line between diagenetic and biogenic endmembers, and therefore argues for the absence of soil contamination^19^. Typical analytical uncertainties on δ^66^Zn are 0.04‰. The cattle and sheep δ^66^Zn values range from 0.85 to 1.09‰ (n = 3). They are higher than the cat (δ^66^Zn = 0.67‰) and the dog (δ^66^Zn = 0.50‰) values, an observation which is in agreement with the trophic level effect observed in previous studies^4,5^. The suckling pig has a δ^66^Zn value similar to that of carnivores (δ^66^Zn = 0.65‰). This observation is not surprising considering that (1) the written record of the hospital documents the feeding of the pigs with leftovers, which included fish and meat^20^ (2) we documented the high trophic level of those pigs in our previous study using C, N and S isotopes^11^. The isotope ranges are consistent with previous observations in other terrestrial mammal food webs^3,4^, as well as the trophic level offset between herbivores and omnivores/carnivores (0.35‰ in Rennes, 0.45‰ in terrestrial Kenyan mammal food web^4^, 0.3‰ in Arctic marine mammal food web^5^). Human δ^66^Zn values are generally lower than those for animals. Individuals showing ^87^Sr/^86^Sr compatible with the local range have significantly lower Zn isotope ratios than those of non-local individuals (Table 2, normal distribution, two-tailed, n = 54, t-test p = 1 × 10^−7^), whose δ^66^Zn values overlap with those of omnivorous/carnivorous mammals (Fig. 3). Contrary to what was previously observed for δ^15^N values, there are no differences between the Zn isotope ratios of men and women. Among the individuals who exhibit ^87^Sr/^86^Sr compatible with the local range, we did not notice differences between socio-economic groups for both δ^15^N or δ^66^Zn values (Table 4). The δ^66^Zn values of the locals from Rennes overlap with values of modern humans from France, whereas non-local individuals have δ^66^Zn values similar to that observed in historical populations from South-Eastern France^7^ (Fig. 3).

**Table 4:** Zn and N isotope ratios of humans exhibiting local Sr isotope ratios depending on the socio-economic group and the historical phase. Phase 1, 2 and 3 are described in the material section.

	δ^66^Zn	2 SD	n	δ^15^N	2 SD	n
**Phase1**						
Non-Privileged (B-B’)	0.39	0.08	2	13.0	—	1
**Phase2**						
Privileged (A, B’)						
Nobles (A’)	0.41	0.02	2	13.5	0.6	2
Non-Privileged (B-B’)	0.35	0.30	4	12.7	1.8	4
Ecclesiastics (C)	0.13	0.10	2	12.4	2.0	2
Soldiers (D)	0.41	0.28	2	12.5	1.8	2
**Phase3**						
Privileged						
A’	0.30	0.34	6	14.1	3.2	3
A-A’	0.30	0.32	16	13.1	2.0	14
B’	0.32	0.24	7	13.1	2.2	7
Ecclesiastics (C)	0.41	0.10	4	13.6	0.4	4

### Correlation between isotope tracers

In order to investigate the influence of different food categories, including marine fish, on the isotope signatures of Sr and Zn, we explored the presence or absence of correlation between these isotope ratios and other dietary proxies (C, N and S stable isotope signature). Among the 54 human teeth analyzed in this study, we previously measured C and N isotope ratios for 47 of them, and S isotope ratios for 29 of them. No correlation has been observed between ^87^Sr/^86^Sr and Sr concentrations, δ^15^N, δ^13^C as well as δ^34^S values. δ^66^Zn values also do not correlate with δ^15^N, δ^13^C and δ^34^S values. A correlation however seems to appear between Zn and N isotope values when animals are taken into account (n = 51, Pearson’s correlation test, R^2^ = 0.21, p = 6 × 10^−4^, Fig. 3B). Sr and Zn isotope values do not correlate when animal and human teeth are considered together (n = 54, Pearson’s correlation test, R^2^ = 0.01, p = 0.60). However, the correlation does exist in human teeth, but only when non-local individuals are included (Fig. 3).

## Discussion

### Geographical origin of Rennes’ humans and animals

Two of the three herbivores analyzed exhibit ^87^Sr/^86^Sr higher than the expected local range according to the IRHUM database^16^ (Fig. 2), but such values exist in the surroundings of Rennes. In agreement with historical evidence, cattle and sheep were probably raised in the countryside, whereas dogs, cats and pigs were urban animals^15,20^ (Fig. 2). The local range defined by all domestic animal Sr isotope signatures therefore represents Rennes and the nearby countryside.

**Figure 4:**
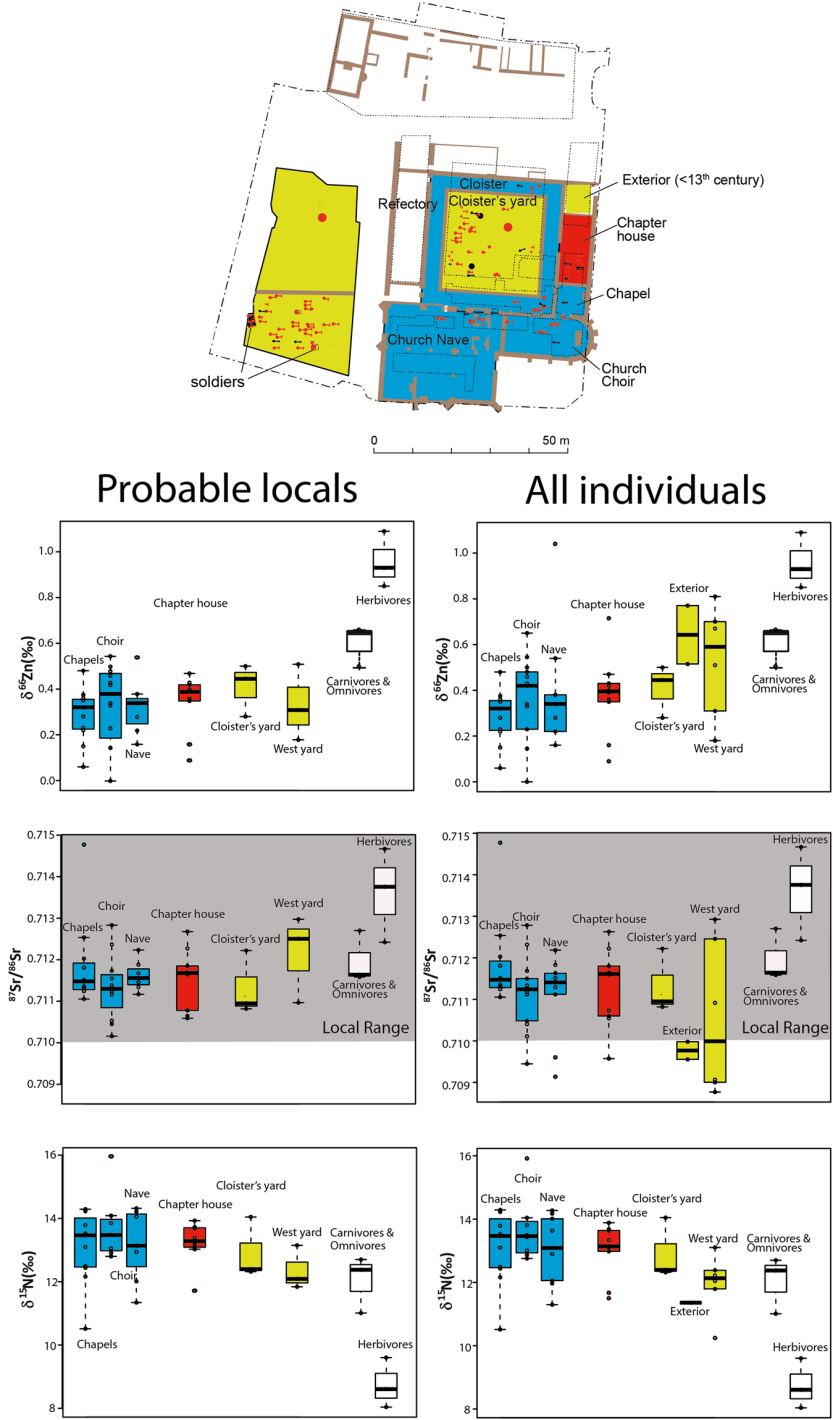
Burial location and associated tooth Sr, Zn and N isotope ratios in teeth of humans buried in the Dominican convent of Rennes and animals from the nearby refuse dump. N isotope ratios were previously published^11^. Blue color: privileged groups, red: ecclesiastics, yellow: commoners. Grey area: range of local ^87^Sr/^86^Sr.The map of the convent was drawn using the software Adobe Illustrator CS6 after the one created using the software Autocad for the excavation reports (Le Cloirec, 2016). The map corresponds to the convent during the 14^th^–16^th^ centuries.

The local ^87^Sr/^86^Sr range defined here is compatible with the one predicted for the whole Armorican Massif, which is also the case for the local S isotope signatures^11^. Individuals exhibiting S and Sr isotope signatures compatible with the local ranges are therefore likely to be originating from Brittany. Individuals showing lower ^87^Sr/^86^Sr isotope signature are likely to come from sedimentary regions, such as the Parisian and Aquitaine Basins, South-Eastern England or South-Eastern France. Strontium isotope data confirm a local origin of women and nobles buried in the Dominican convent, as well as the presence of non-local individuals among the medieval ecclesiastics, commoners and soldiers buried in mass graves (Fig. 4). Two individuals buried in the church have high ^87^Sr/^86^Sr isotope signatures but non-coastal δ^34^S values, suggesting they have spent their childhood out of Brittany.

### Zn isotope ratios: a promising new dietary tracer?

**Figure 5:**
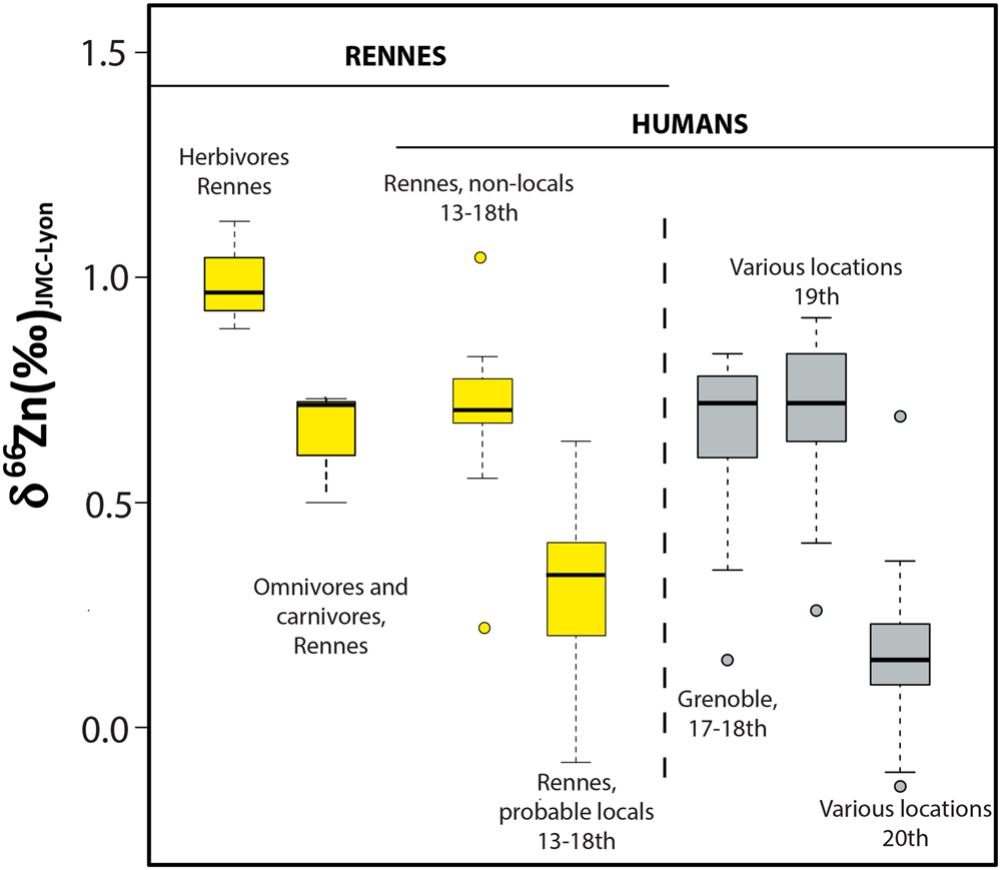
Zn isotope ratios in teeth of animals and humans from Rennes (this study, yellow boxplots) and from various locations in France (grey boxplots, data from Jaouen *et al*. 2017).

The range of δ^66^Zn values measured in teeth showing Sr isotope ratios compatible with local signatures overlaps with that of modern (20^th^ century) individuals but strongly differs from previous historical periods (17^th^ to 19^th^ centuries) (Fig. 5). However, individuals exhibiting Sr isotope signatures compatible with other rock types such as volcanic or sedimentary rocks - that is to say lower than the local range - have similar Zn isotope ratios to the abovementioned historical populations. A large part of the preindustrial teeth previously studied^7^ belonged to individuals coming from sedimentary regions (Supplementary Information 1). The Zn isotope composition of igneous rocks and clastic sediments is fairly constant (0.2 < δ^66^Zn < 0.4‰)^13^, but siliceous and calcareous sediments can show higher Zn isotope ratios^13,21^, especially in limestones, for which the δ^66^Zn values can reach up to 1.35‰^22,23^. To verify if the Zn isotope differences between individuals showing high ^87^Sr/^86^Sr and those having low ^87^Sr/^86^Sr ratios could be simply related to the Zn isotope composition of the bedrock, we compared the Zn isotope ratios of all human historical teeth from this study and a previous one on Zn isotope ratios in teeth of French individuals^7^, to the expected values of the associated bedrock (Supplementary Information 1). We found that humans coming from regions with chalky bedrock indeed exhibited the highest Zn isotope ratios, but were not significantly different from the humans coming from regions with igneous or sandstone bedrocks. Moreover, individuals coming from igneous inland regions still had higher δ^66^Zn values than the local Bretons from this study (Supplementary Information 1). If the geology can have an impact on local human teeth ratios^3,4^, it seems that biological processes in soils tend to overprint these original ratios^24^.

Based on the absence of relationship between Zn isotope ratios of teeth and geology, as well as the established difference between δ^66^Zn values of herbivores and omnivores/carnivores^1,2,4^, diet is the most likely factor to account for the peculiar isotope signature of the local Bretons from Rennes. The δ^66^Zn_tooth_ values of the humans are clearly lower than that of the associated fauna, including the cat and the dog, and overlap those of modern individuals (carnivores, Figs 3, 5). As mentioned above, this low Zn isotope ratio could be the signature of a substantial animal product consumption of high trophic level, which would explain the lower Zn isotope signatures than the local carnivores. The French historical populations, which have been previously studied, characterized by high Zn isotope ratios, were workers coming from inland regions (e.g. French Alps, Rhone Valley, Jura, Pyrenees, Vosges), with limited access to fish consumption^7,25^. Whereas, Rennes’s individuals are likely to have had a frequent meat and fish consumption: nobles and middle class to display their wealth, ecclesiastics to respect fasting rules, but also urban workers who were complaining of too much salted fish consumption^15^. The Bretons were also known to have a substantial meat consumption relative to human populations from other French regions^12^. Therefore, high δ^66^Zn values of non-local individuals, mostly buried out of the convent walls and therefore likely to be commoners, could be explained by a cereal-based diet. Cereals, as most plant foods, have indeed much higher δ^66^Zn values than animal products^2^, and were the main dietary source of the non-privileged French population of that time^12^, especially outside of Brittany^15^. To explain the fact that Rennes humans exhibit lower Zn isotope ratios than dogs and cats, the existence of high trophic level food consumption, namely carnivorous fishes and/or suckling pigs, must be invoked. The pigs from the refuse dump of Rennes were bred in a hospital yard and fed with leftovers which daily included meat and fish^20^. Zinc isotope studies in archeological contexts being in their infancy, it is not possible to say at this stage if different signatures of freshwater or marine fish consumption can be expected. The absence of a correlation between carbon isotope ratios and ^87^Sr/^86^Sr values documents a weak contribution- albeit existing, according to zooarcheological evidence - of marine fish in Rennes human diets. We documented previously very high δ^15^N values in tooth and bone collagen of humans and animals, and could not conclude if these high N isotope ratios were more due to the consumption of herbivorous mammals, suckling animals or eels^11^. Given the fact that eels are migratory aquatic organisms and constitute 45% of the fish remains found in the refuse midden close to the convent as well as the refectory soil^20,26^ and that suckling pigs were a sought-after food at that time^11^, the Zn isotope ratios could reflect the signature of a substantial eel and pork consumption. Animal product consumption in Rennes’ diets is in every instance more important than in those of the historical French populations previously studied^12,15,16,25,26^, which explains the low Zn isotope ratios.

### Absence of correlation between dietary tracers

If both Zn isotope ratios and N isotope ratios are indicators of the trophic level effect, one should expect a correlation between these two tracers. This correlation exists when all species are considered together (Fig. 3B), but is absent among human values. This pattern has already been observed in marine mammals^5^: an interspecific correlation between δ^66^Zn and δ^15^N values was reported but did not exist between individuals from the same species. Zn isotope ratios were measured in tooth enamel, whereas N isotope ratios were measured in the dentine. These two dental hard tissues with different formation times could therefore record different diets. We however reject a change of diet between the time of formation of the dental enamel and dentine sampled as being the explanation of the absence of correlation between δ^66^Zn and δ^15^N values, since the lack of correlation has already been reported for bones and blood^18,27^.

The two isotope tracers are therefore both influenced by trophic level, but other dietary factors may influence one of the isotope ratios and not the other one. For example, N isotope ratios, measured in collagen, mostly provide information on the protein portion of the diet^28^, whereas Zn, measured in bioapatite, is likely to reflect the bulk diet – albeit mostly the animal product portion. A trophic level effect is also observed in breastfeeding individuals^29^ for N isotope signatures but is not expected for Zn isotopes: the M1 milk teeth - which form their enamel during the first year of life - that we measured in this study, and that has also been previously analyzed^7^ did not differ from M2 and M3 permanent teeth. Moreover, milk products do not show depleted Zn isotope signatures compared to meat or fish^2^. Finally, environmental factors are likely to differentially influence the local Zn and N isotope signatures of the soils and plants^4,9,13,30,31^, which could account for the lack of correlation between these two dietary indicators.

### Diet, mobility and social status

Colleter *et al*.^11^ reported dietary differences related to social status for N isotopes, although they were more obvious among adults (bone values) than during childhood (dentine values). When the whole population is considered together, dietary and mobility differences can also be observed for Zn isotopes relative to the burial location: individuals buried in the church and the chapels (groups A and B’) are, in general, more local and to also have a diet richer in animal products (according to δ^66^Zn and δ^15^N values, Figs 3 and 4) than individuals buried in the exteriors. However, when only individuals with local ^87^Sr/^86^Sr ratios are considered, the isotope differences between burial locations disappear for δ^66^Zn and δ^15^N values (Fig. 4, Table 2). In the present study, most of the non-local individuals were buried in the exteriors. It is therefore difficult to know whether social status isotope differences are due to dietary or environmental factors, or if it is related to a sample size bias. Nevertheless, as mentioned before, a substantial animal product consumption of the urban workers, likely to be buried in the exteriors, is consistent with historical writings^15^. A gender difference is clearly existing for N isotopes in teeth^11^ but not for Zn isotopes. A female diet including a significant proportion of young animals but a similar amount of animal products relative to the male diet could explain such an isotopic pattern.

## Conclusions

The Zn stable isotope compositions measured in the teeth of individuals buried in the Dominican convent of Rennes show a remarkable pattern: (1) local privileged individuals exhibit overlapping values with modern individuals from France, which could be explained by substantial meat and/or fish consumption (2) individuals identified as migrants using Sr isotopes have Zn isotope ratios similar to those of poor French individuals previously analyzed from the 17^th^ to the 19^th^ centuries. This is partially explained by a limited influence of the geology on the Zn isotope composition of food products eaten during the childhood combined with a reduced meat consumption of migrating individuals. Local Rennes humans exhibit lower Zn isotope ratios than the associated fauna, including carnivores, which can possibly be explained by carnivorous fish and pork consumption. Fasting rules indeed imposed the consumption of fish a day out of three in medieval and early modern Western Europe. Given the historical, zooarcheological and other isotope data obtained from Rennes Dominican’s convent, this carnivorous fish consumption could mostly consist of codfish and eels. Zn isotopes have therefore a strong potential to trace the consumption of high trophic level food, and potentially fish, in ancient human diets.

## Methods

### Material

Rescue excavations in the city center of Rennes recently permitted the study of the implantation and evolution of a mendicant convent which has been described by Le Cloirec (2016)^26^. The Dominican convent was founded outside the walls of the city in 1368. Between the end of the 14^th^ and the 18^th^ century, the settlement was an important place of pilgrimage – because of the presence of a “miraculous painting” in the convent - and burial, especially for the parliamentary nobility^14,15,32^. A multi-isotope study previously documented the diet of this population^11^. Three phases of burial are differentiated on the site. The first phase (13^th^ c., phase 1) predates the construction of the convent. The second (phase 2) goes from the end of the 14^th^ century to the 16^th^ century. The last period (phase 3) covers the 17^th^ and 18^th^ centuries and therefore corresponds to the modern period. Additional information is available in the Table S1 of the Supplementary data. In total, 54 human molars (M2 and M3) were sampled. The faunal remains (16^th^ century, end of the phase 2, Fig. 1) consist in the most common species found in the refuse midden of the hospital concomitant and contemporaneous to the convent.

### Methods

All the analyses were conducted in the laboratories of the Department of Human Evolution at the Max Planck Institute for Evolutionary Anthropology (MPI-EVA) in Leipzig, Germany, in accordance with approved guidelines and regulations.

The samples were mechanically cleaned using a dental drill equipped with a diamond tip. Two small pieces were sampled (5–20 mg). Dentine was removed with a diamond-tipped burr. For Sr isotope analyses, samples were digested in nitric acid and purified using the ion exchange method described in Maréchal *et al*. (1999)^33^. Each batch of preparation included 13 samples. One blank and one external standard SRM 1486 (bone meal) were analyzed to verify that no contamination or purification problem happened during the column chromatography. For Zn isotope analyses, samples were digested in hydrochloric acid (HCl), evaporated and dissolved in hydrobromic acid (HBr, 1.5 M). Each batch of preparation included a blank and an external standard (in house standard AZE bone powder and/or the SRM 1400 bone ash, Table S4). Zinc was then purified using a protocol adapted from Moynier *et al*. (2006)^34^ previously described in Jaouen *et al*. (2016b)^5^. The Sr and Zn isotope analyses were conducted using a Thermo Fisher Neptune MC-ICP-MS at the Max Planck Institute for Evolutionary Anthropology (Leipzig. Germany). δ^66^Zn values are expressed relative to the standard JMC-Lyon. External reproducibility is 0.04‰ for δ^66^Zn values and 0.000024 for Sr ratios (1 SD). The protocol followed for isotope and concentration analyses was previously described for Sr^35,36^ and for Zn^5^.

### Data availability statement

All data generated or analyzed during this study are included in this published article and the Supporting Information file.

## Electronic supplementary material

Supplementary Information
