# Supplementary material for: Tracing intensive fish and meat consumption using Zn isotope ratios: evidence from a historical Breton population (Rennes, France)
Source: Sci Rep. 2018 Mar 22;8:5077. doi: 10.1038/s41598-018-23249-x (PMC5864724; doi:10.1038/s41598-018-23249-x)
Supplement: Supplementary file 1 — Supplementary Information [file 41598_2018_23249_MOESM1_ESM.docx]

**Supplementary Information of the manuscript**

**Tracing intensive fish and meat consumption using Zn isotope ratios: evidence from a historical Breton population (Rennes, France).**

***Authored by***

Klervia Jaouen, Rozenn Colleter, Anita Pietrzak, Marie-Laure Pons, Benoît Clavel, Norbert Telmon, Éric Crubézy, Jean-Jacques Hublin, Michael P. Richards.

**Supplementary Information 1**

**Relationship between the geological context and the Zn isotope ratios of human teeth**

**Introduction**

Igneous rocks exhibit very homogenous δ^66^Zn values (0.3 ± 0.1 ‰, ^1,2^, whereas sedimentary rocks can exhibit a broader range of δ^66^Zn values ^1–3^ with the highest values measured in carbonates (up to 1.4 ‰, ^4, 5^). Local geology might therefore impact local isotope ratios of the food web. The geographical origin of the individuals in Rennes was therefore assessed using Sr isotopes and previously published S isotope ratios (Colleter et al., In press).

Historical Brittany geographically corresponds to the Armorican Massif which formed during the Cadomian and Hercynian orogenies. The ^87^Sr/^86^Sr ratios are therefore generally high (^87^Sr/^86^Sr>0.71). Expected ranges can be found in the IRHUM database^6^ and are reported in the Figure 1. We did not analyze local plants to examine the Sr isotope compositions of areas surrounding the convent, because it is located in the city center of Rennes and the soil values would therefore not reflect the medieval context. We however examine the Sr isotope ratios of the medieval animal dental enamel: the cattle and the sheep are likely to come from close-by areas (immediate countryside); Pigs, cats and dogs are urban animals ^7,8^.

Strontium isotope ratios of human dental enamel could also be impacted by marine food consumption, characterized by the Sr isotope signature of modern seawater (^87^Sr/^86^Sr =0.7092), less radiogenic than the local geology. The presence of a correlation between δ^13^C values and ^87^Sr/^86^Sr in human teeth reveals the impact of marine food consumption on ^87^Sr/^86^Sr_tooth_. This contribution of marine food into the diet of Rennes Dominican’s convent individuals is however known to be minor according to C isotope ratios measured in their bone and tooth collagen (Colleter et al., in press).

Additionally, the S isotope analyses we conducted in our previous study (Colleter et al., in press) help constraining the geographical origin of each individual. In this previous study, we demonstrated that S isotope ratios of local animals and individuals corresponded to coastal values. Brittany is a peninsula with an oceanic climate. Consequently, we hypothesized that marine influence δ^34^S values might be generally observed anywhere in Brittany. Non-coastal values were therefore interpreted as the signature of a living place located outside of Brittany and far from the coast.

The geographical origin has therefore been documented to control for the impact of the geology on the local Zn isotope signature of teeth. However, previous documentation of Zn isotope ratios of human teeth from various location argue for a small impact of the bedrock on the local human values, as discussed below.

**Role of local geological settings and soils on the Zn isotope composition in teeth:**

The Zn isotope composition of a bulk plant depends mostly on two parameters: the fractionation that occurs between the plant and the soil in the rhizosphere during zinc uptake by the roots (e.g. 9, 10) and the initial δ^66^Zn of the soil (e.g. 11, 12). The latter is in turn controlled by the nature of the bedrock and the pedogenetic processes (2 and references therein). To understand the Zn isotope composition in human teeth and to decipher the diet contribution to the signal, it is therefore crucial to unravel the role of geology and soil pedogenesis on the Zn isotopes distribution.

***Zinc isotopes in terrestrial bedrocks****.*

In the past 15 years, Zn isotopes have been investigated in terrestrial rocks, which show a large distribution, with values ranging from ~ -0.6 ‰ in some ultramafic rocks ^13^ to ~ +1.5 ‰ in carbonates (5). Bedrocks relevant to the study, i.e. in France or the south part of England, are: igneous rocks (e.g. granites), metamorphic rocks (e.g. gneisses), clastic sediments (e.g. shales) and carbonate units (e.g. limestones). Igneous rocks and clastic sediments – including loess, shales and dust – show a very narrow range for Zn isotope compositions, with δ^66^Zn = 0.31 ± 0.24 ‰ and δ^66^Zn = 0.28 ± 0.26 ‰, respectively (2 s.d., 2 and references therein). So far, little is known on the impact of metasomatic and metamorphic processes on the Zn isotope composition of rocks, but recent studies tend to show that these processes induce limited Zn isotope fractionation (< 0.2 ‰), usually in favour of an enrichment in light Zn isotopes ^14,15^. Igneous and clastic-derived metamorphic rocks should therefore display δ^66^Zn values of ~0 to 0.4 ‰ (see supplementary table S1). Carbonates show the wider distribution, with δ^66^Zn from ~0.35 to 1.35 ‰, for an average composition of ~0.9 ‰ (2 and references therein), which is significantly higher than igneous and igneous-derived rocks. Parts of France with limestone as the dominant bedrock (e.g. carbonates units in large sedimentary basin such as the Parisian Basin) are therefore expected to show ^66^Zn-enriched bedrock composition compared to old crystalline massifs (such as the Armorican Massif in Brittany) or Quaternary formations (e.g. fluvio-glacial deposits, loess).


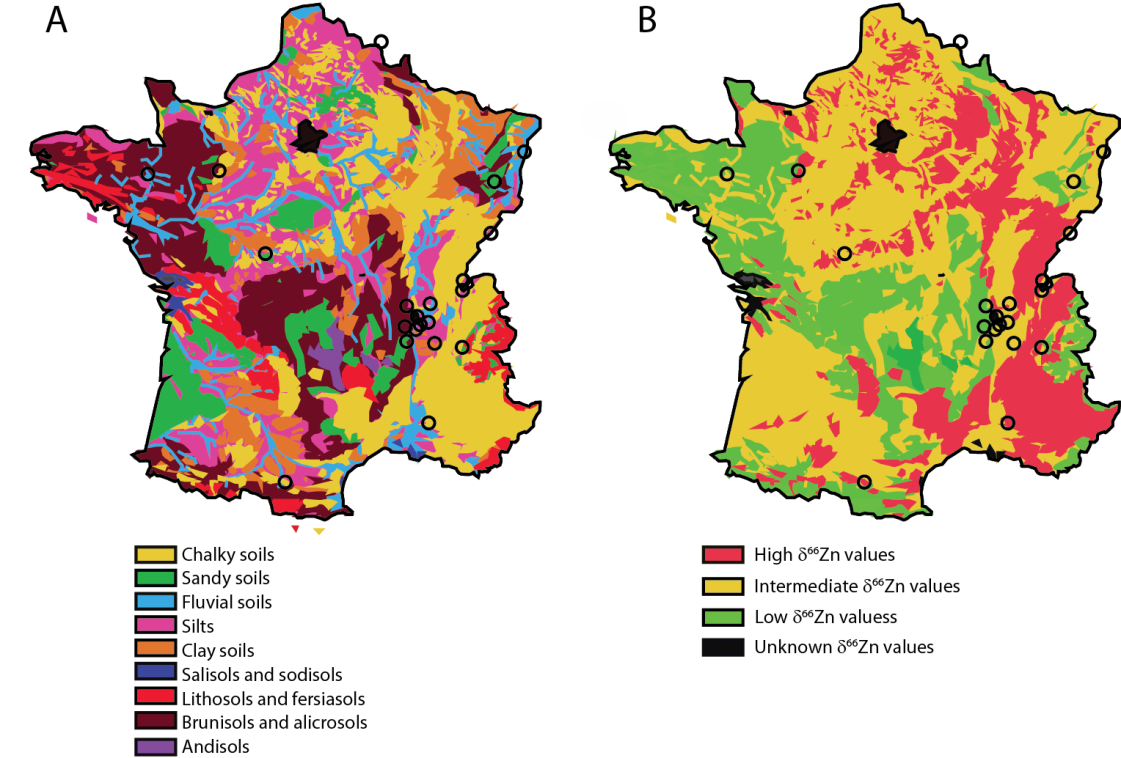


**Figure S1:** Simplified map of soils in France and expected associated Zn isotope ratios. (blanc map fron Daniel Dalet, <http://www.histgeo.ac-aix-marseille.fr)>**.** The map on the right with estimated δ^66^Zn values based on the various measurements published so far on different type of soils (see text for details). Open circles represent the geographical origin of the individuals for which Zn isotope data are available.

***Zinc isotopes in soils.***

In a recent study by Fekiacova et al. (12), the Zn isotope composition of a various types of polluted and unpolluted soils has been investigated. The study shows that the overall range of δ^66^Zn values in soils is smaller than that of terrestrial rocks, going from ~ -0.53 to 0.63 ‰ (12 and references therein). Unpolluted soils display even smaller variations, with a δ^66^Zn distribution centered at 0.2 ‰. The pedogenetic processes seem to smooth out the bedrock δ^66^Zn differences.

***Relationships between* δ^66^Zn *in human teeth and geographical origin – data from Jaouen et al., (17).***

In a previous study by Jaouen et al., ^16^, Zn isotope in teeth from various individuals from the preindustrial period have been analysed (17). These samples from the late 19^th^ century are well characterized, in particular regarding the birthplace and origin of the individuals. The Supplementary Table S1 summarize the origin, sex, age at death, time of death, and the Zn isotope composition of the teeth analysed (data from 17). The professional activity of the individuals was also documented: the samples individuals were from the working class and were likely to have had a cereal-based diet. For each individual, we have determined the geological context of the place they originated from, using the 1:50 000 geological maps of France from the BRGM (available online at: <http://infoterre.brgm.fr/viewer/MainTileForward.do>), to decipher the potential link between the δ^66^Zn and the local bedrocks. These geological settings have been reported in the Supplementary Table S1. Four main geological settings have been identified, and the individuals have been assigned to four different groups accordingly, people born and raised in localities: (1) dominated by limestone, (2) with the presence of limestones, (3) metamorphic units and (4) Quaternary formations (e.g. alluvium, fluvio/glacio-deposits, silts). The expected δ^66^Zn of the bedrocks for these 4 groups have been reported in the Supplementary Table S1, as well as the average Zn isotope compositions (+2 s.d.) of the groups. For groups (1) and (2), where the highest δ^66^Zn in the bedrocks are expected due to the presence of carbonates, the populations show heavy Zn isotope compositions with average values of 0.77 ± 0.20 ‰ and 0.76 ± 0.21 ‰ respectively. These two groups show identical δ^66^Zn values and distribution, despite the scarcity of carbonates in group (2) compared to group (1). These two groups also overlap with the values measured for individuals of groups (3) and (4), for which the bedrock δ^66^Zn is expected to be much lower (average δ^66^Zn of ~0.20 ‰ compared to ~0.90 ‰ for limestones), and none of the four groups is statistically different from any other (Student’s t-test, 95 C.I.). These results suggest that the geological setting play a very limited role on the Zn isotope composition of human teeth and that the bedrock δ^66^Zn signatures are overprinted by biological processes, such as trophic fractionation. For information, the dominant soils (see Supplementary Figure S1, A) have also been reported for each group in the Supplementary Table S1.

|  | Id | City | Department | Death | Tooth | Enamel |  | Geological setting | Expected δ^66^Zn (‰) values for the bedrock | Main type of soils |
| --- | --- | --- | --- | --- | --- | --- | --- | --- | --- | --- |
|  |  |  |  |  |  | δ^66^Zn (‰) |  |  |  |  |
| Group 1 - limestones | CCEC | Luisans | Doubs | 1877 | M^3^L | 0.65 |  | Limestones |  |  |
|  | CCEC | Chatel de Joux | Jura | 1877 | M^3^L | 0.85 |  | Limestones |  |  |
|  | CCEC | Baumes les Dames | Doubs | 1879 | M_3_L | 0.71 |  | Limestones |  |  |
|  | CCEC | Monleret | Isère | 1877 | M_3_R | 0.85 |  | Limestones |  |  |
|  |  |  |  |  |  | **average** | **2 s.d.** |  | 0.3 - 1.4 ‰ | chalky soils |
|  |  |  |  |  |  | **0.77** | **0.20** |  | average: ~0.9 ‰ |  |
|  |  |  |  |  |  |  |  |  |  |  |
| Group 2 - presence of limestones | CCEC | Grilly s/Gey | Ain | 1877 | M_3_R | 0.74 |  | Mostly moraines, some shales and some limestones at ~5 km. |  |  |
|  | CCEC | Artix | Arriège | 1879 | M_3_L | 0.63 |  | Pouddingstones. Marnes, some moraines and some limestones |  |  |
|  | CCEC | La Balme | Isère | 1879 | M^3^L | 0.79 |  | Mostly alluviums, moraines and some limestones |  |  |
|  | CCEC | Sermérieu | Isère | - | M_3_L | 0.88 |  | Mostly glacio-fluvial deposits, moraines, sandstones, a very small fraction of limestones |  |  |
|  |  |  |  |  |  | **average** | **2 s.d.** |  | 0.2 - 0.8 ‰* | fluvial/sandy/chalky/clay soils, silts |
|  |  |  |  |  |  | **0.76** | **0.21** |  | * |  |
|  |  |  |  |  |  |  |  |  |  |  |
| Group 3 - metamorphic units (Hercynian orogeny) | CCEC | Thurins | Rhône |  | M^3^L | 0.41 |  | Mont-du-Lyonnais metamorphic complex (gneiss, schists) + granites |  |  |
|  | CCEC | St Chamond | Loire | 1877 | M_3_L | 0.56 |  | Metamorphic terrains: gneiss, schists, micaschists |  |  |
|  | CCEC | Tarare | Rhône | 1879 | M_3_L | 0.71 |  | Mont-du-Lyonnais metamorphic complex (gneiss, schists) + metasediments |  |  |
|  | CCEC | Tarare | Rhône | 1877 | M^3^R | 0.58 |  | Mont-du-Lyonnais metamorphic complex (gneiss, schists) + metasediments |  |  |
|  | CCEC | Vienne | Isère | 1881 | M_3_R | 0.74 |  |  | 0.0 - 0.4 ‰ | brunisol and alicrosols, sandy soils |
|  |  |  |  |  |  | **average** | **2 s.d.** |  | average: ~0.2 ‰ |  |
|  |  |  |  |  |  | **0.60** | **0.26** |  |  |  |
|  |  |  |  |  |  |  |  |  |  |  |
| Group 4 - quaternary formations | CCEC | Strasbourg | Bas Rhin | 1877 | M^3^L | 0.64 |  | Loess |  |  |
|  | CCEC | Lyon | Rhône | 1878 | M_3_R | 0.81 |  | Mostly glacio-fluvial deposits, moraines. alluvium and loess |  |  |
|  | CCEC | Fresnay | Sarthe | 1879 | M^3^R | 0,73 |  | Mostly silicious silts + flints, clay |  |  |
|  | CCEC | Beaurepaire | Isère | 1879 | M_2_ | 0.91 |  | Silts, clay, moraines, alluvium |  |  |
|  | CCEC | Védène | Vaucluse | 1879 | M_3_R | 0.85 |  | Alluvium, colluvium, lake deposits |  |  |
|  | CCEC | Vesoul | Haute-Saône | 1879 | M^3^L | 0.85 |  | Mostly alluvium and Toarcian bituminous schists |  |  |
|  | CCEC | Lyon | Rhône | 1879 | M^3^L | 0.65 |  | Mostly glacio-fluvial deposits, moraines, alluvium and loess |  |  |
|  | CCEC | Lyon | Rhône | 1877 | M_3_L | 0.61 |  | Mostly glacio-fluvial deposits, moraines, alluvium and loess |  |  |
|  | CCEC | Le Tholy | Vosges | 1881 | M_3_L | 0.26 |  | Mostly glacial deposits, moraines + granites |  |  |
|  |  |  |  |  |  | **average** | **2 s.d.** |  | 0.15 - 0.35 ‰ | silts, fluvial and clay soils |
|  |  |  |  |  |  | **0.70** | **0.39** |  | average: ~0.3 ‰ |  |
| Locals, Group 2 | Saint-Laurent | Grenoble | Isère | 17-18th | M2 and M3 | **average** | **2 s.d.** | Mostly alluviums, moraines and some limestones | 0.2 - 0.8 ‰* | fluvial/sandy/chalky/clay soils, silts |
|  |  |  |  |  |  | **0.66** | **0.34** |  |  |  |
| Locals, Group 3 | Brittany | Rennes | Ille-et-Vilaine | 13th-18th | M2 and M3 | **average 0.33** | **2 s.d. 0.28** | Metamorphic and igneous terrains: gneiss, schists, micaschists, granites + Fluvial deposits | 0.0 - 0.4 ‰ | brunisol and alicrosols, lithosols and fersiasols, sandy soils, silts, clay and fluvial soils |

Table S1: Zn isotope ratios of human teeth and associated bedrock from their geographical origin. Data for CCEC and Saint-Laurent population (* symbol) come from the reference 17. Data for Brittany comes from this study.


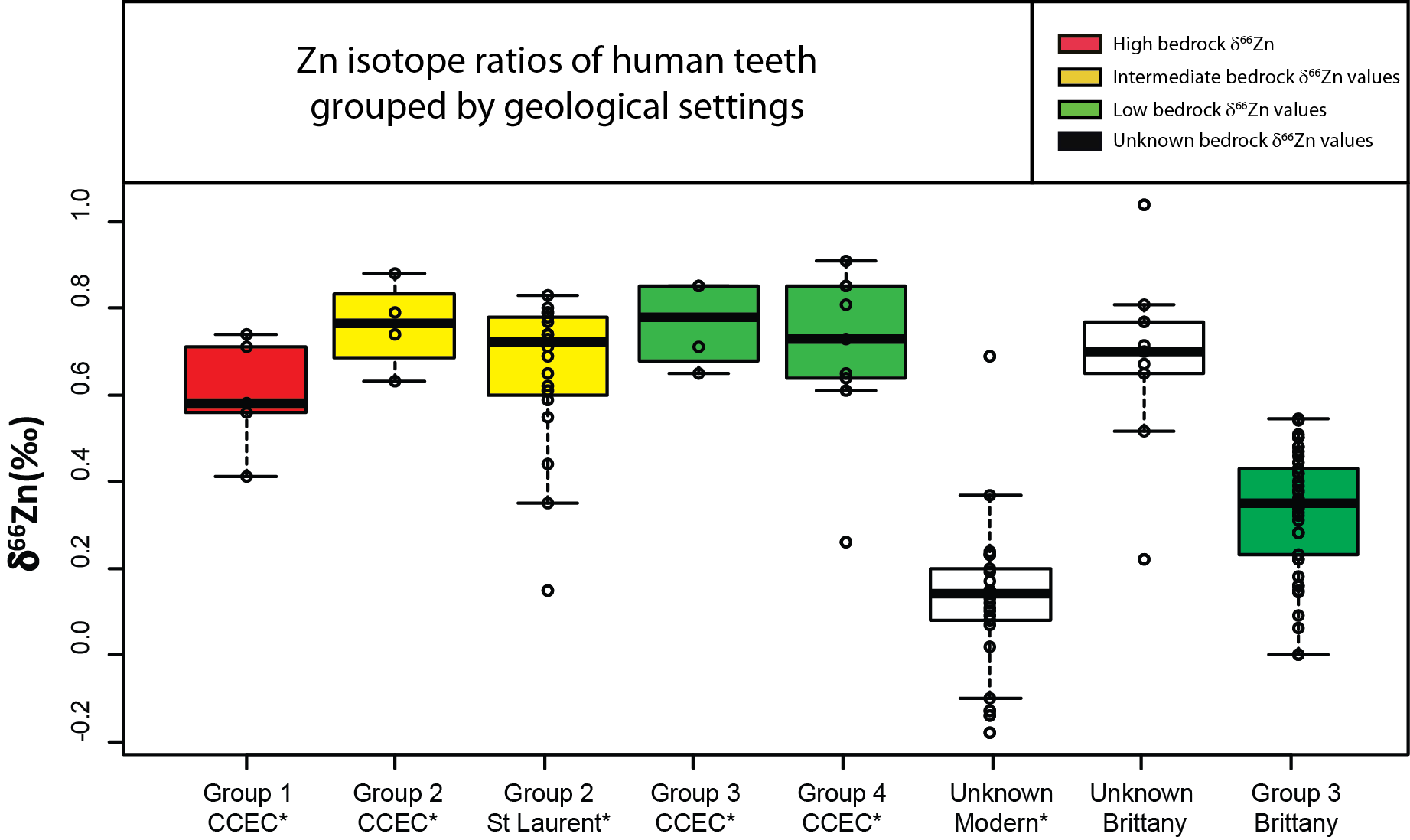


**Figure S2**: Zn isotope ratios of human teeth grouped by geological settings. The groups 1, 2, 3 and 4 are described in the Table S1.* data from Jaouen et al. (17). St-Laurent: early modern population from Grenoble, French Alps. CCEC: 19^th^ century individuals from various French locations and from the working class. Modern: modern human teeth from various locations. France. Brittany: data from this study.

***Geological settings and soils in pre-industrial Brittany: contribution to the* δ^66^Zn *signal in human teeth*.**

Historical Brittany geographically corresponds to the Armorican Massif which formed during the Cadomian and Hercynian orogenies. Its geological setting is mostly dominated by (i) metamorphic and igneous terrains: gneiss, schists, micaschists, granites; and (ii) Quaternary formations: fluvial deposits (see Supplementary Table S1). This setting is very similar to the ones of groups (3) and (4) from Supplementary Table S1. The variety of soils found in Brittany (see Supplementary Figure S1, A and Supplementary Table S1) is also comparable to the ones described for these two groups. However, the individuals identified by their high ^87^Sr/^86^Sr as locals, born and raised in Brittany, display tooth Zn isotope compositions that are significantly lower (Student’s t-test, p<1.10^-9^, 95 C.I.) than that of groups (3) and (4), with average δ^66^Zn of 0.33 ± 0.28 ‰ for locals Bretons versus average δ^66^Zn of 0.60 ± 0.26 ‰ and 0.70 ± 0.39 ‰ for groups (3) and (4) respectively. This observation provides strong evidence that the Brittany geological setting is not responsible for the low δ^66^Zn values measured in Bretons’ teeth, and that this low Zn isotope signature reflects a difference in diet between the Bretons and the previously studied preindustrial populations.

**References**

1. Cloquet, C., Carignan, J., Lehmann, M. F. & Vanhaecke, F. Variation in the isotopic composition of zinc in the natural environment and the use of zinc isotopes in biogeosciences: a review. *Anal. Bioanal. Chem.* **390,** 451–463 (2008).

2. Moynier, F., Vance, D., Fujii, T. & Savage, P. The isotope geochemistry of zinc and copper. *Rev. Mineral. Geochem.* **82,** 543–600 (2017).

3. Maréchal, C. N., Nicolas, E., Douchet, C. & Albarède, F. Abundance of zinc isotopes as a marine biogeochemical tracer. *Geochem. Geophys. Geosystems* **1,** (2000).

4. Luck, J. M., Ben, O. D., Albarede, F. & Telouk, P. Zn and Cu Isotopes Tracers of Metal Origin in the Dissolved and Particulate Loads of Rain. *Geochim Cosmochim Acta A* **7619,** (1999).

5. Pichat, S., Douchet, C. & Albarède, F. Zinc isotope variations in deep-sea carbonates from the eastern equatorial Pacific over the last 175 ka. *Earth Planet. Sci. Lett.* **210,** 167–178 (2003).

6. Willmes, M. *et al.* The IRHUM (Isotopic Reconstruction of Human Migration) database. *Earth Syst. Sci. Data* **6,** 117 (2014).

7. Croix, A. *La Bretagne aux 16e et 17e siècles: la vie, la mort, la foi*. **2,** (Maloine, 1981).

8. Clavel, B. Données archéozoologiques et fouilles d’hôpitaux : l’exemple de l’hôpital sainte Anne (Rennes, Ille-et-Vilaine). in *Les établissements hospitaliers en France du Moyen Âge au XIXe siècle* (Le Clech-Charton S., 2010).

9. Weiss, D. J. *et al.* Isotopic discrimination of zinc in higher plants. *New Phytol.* **165,** 703–710 (2005).

10. Aucour, A. M., Pichat, S., Macnair, M. R. & Oger, P. Fractionation of Stable Zinc Isotopes in the Zinc Hyperaccumulator Arabidopsis halleri and Nonaccumulator Arabidopsis petraea. *Environ. Sci. Technol.* **45,** 9212–9217 (2011).

11. Viers, J. *et al.* Evidence of Zn isotopic fractionation in a soil–plant system of a pristine tropical watershed (Nsimi, Cameroon). *Chem. Geol.* **239,** 124–137 (2007).

12. Fekiacova, Z., Cornu, S. & Pichat, S. Tracing contamination sources in soils with Cu and Zn isotopic ratios. *Sci. Total Environ.* **517,** 96–105 (2015).

13. Pons, M.-L. *et al.* Early Archean serpentine mud volcanoes at Isua, Greenland, as a niche for early life. *Proc. Natl. Acad. Sci.* **108,** 17639–17643 (2011).

14. Pons, M.-L., Debret, B., Bouilhol, P., Delacour, A. & Williams, H. Zinc isotope evidence for sulfate-rich fluid transfer across subduction zones. *Nat. Commun.* **7,** (2016).

15. Inglis, E. C. *et al.* The behaviour of iron and zinc stable isotopes accompanying the subduction of mafic oceanic crust: A case study from Western Alpine Ophiolites. *Geochem. Geophys. Geosystems* (2017).

16. Jaouen, K., Herrscher, E. & Balter, V. Copper and zinc isotope ratios in human bone and enamel. *Am. J. Phys. Anthropol.* **162,** 491–500 (2017).

**Supplementary Information 2.**

**Additional tables**

| **ID SEP** | **INDIV US** | **Tooth** | **δ^66^Zn_1_** | **SD_A_** | **δ ^66^Zn2** | **δ ^66^Zn_t_** | **SD_I_** | **Zn (ppm)** | **^87^Sr/^86^Sr** | **Sr (ppm)** | **δ ^13^C_t_** | **SD** | **δ ^15^N_t_** | **SD** |
| --- | --- | --- | --- | --- | --- | --- | --- | --- | --- | --- | --- | --- | --- | --- |
| 308 | 20134 | M3_inf_ R | 0.67 |  |  | 0.67 |  | 108 | 0.708767 | 15 | -19.14 | 0.03 | 12.22 | 0.41 |
| 321 | 20180 | M2_inf_ R | 0.18 | - |  | 0.18 |  | 213 | 0.712926 | 124 | -19.23 | 0.08 | 12.04 | 0.04 |
| 329 | 20211 | M3_inf_ R | 0.81 |  |  | 0.81 |  | 123 | 0.709067 | 75 | -19.45 | 0.15 | 12.38 | 0.03 |
| 337 | 20787 | M3_inf_ R | 0.31 |  |  | 0.31 |  | 92 | 0.712455 | 56 | -19.14 | 0.04 | 11.80 | 0.04 |
| 337 | 20788 | M3_inf_ R | 0.51 |  |  | 0.51 |  | 104 | 0.710915 | 23 | -19.10 | 0.01 | 13.11 | 0.04 |
| 337 | 20801 | M3_sup_ L | 0.70 |  |  | 0.70 |  | 125 | 0.709002 | 34 | -19.39 | 0.10 | 10.25 | 0.04 |
| 2 | 50096 | M3_inf_ R | 0.50 |  |  | 0.50 |  | 197 | 0.710818 | 88 | -18.90 | 0.06 | 14.05 | 0.10 |
| 13 | 50148 | M3_inf_ L | 0.41 | - | 0.48 | 0.45 | 0.05 | 128 | 0.710950 | 53 | -20.02 | 0.01 | 12.40 | 0.05 |
| 26 | 51380 | M3_inf_ L | 0.28 |  |  | 0.28 |  | 168 | 0.712221 | 104 | -19.81 | 0.05 | 12.32 | 0.03 |
| 55 | 60647 | M2_inf_ L | 0.28 |  |  | 0.28 |  | 142 | 0.714771 | 112 | -19.99 | 0.07 | 10.51 | 0.07 |
| 64 | 60699 | M2_inf_ L | 0.38 | 0.02 |  | 0.38 |  | 149 | 0.711053 | 182 | -19.30 | 0.11 | 14.27 | 0.01 |
| 136 | 60983 | M3_inf_ R | 0.36 | - |  | 0.36 |  | 300 | 0.711359 | 121 | -18.51 | 0.02 | 13.53 | 0.10 |
| 162 | 61087 | M2_sup_ L | 0.23 |  |  | 0.23 |  | 48 | 0.712537 | 88 | -19.53 | - | 12.49 | - |
| 179 | 61163 | M2_inf_ L | 0.15 |  |  | 0.15 |  | 91 | 0.711243 | 96 | -19.15 | - | 13.47 | - |
| 235 | 61534 | M3_sup_ L | 0.32 |  |  | 0.32 |  | 117 | 0.712028 | 65 | -18.77 | 0.05 | 13.10 | 0.04 |
| 258 | 61739 | M3_sup_ R | 0.22 |  |  | 0.22 |  | 142 | 0.711475 | 119.3 | -19.40 | 0.02 | 14.29 | 0.17 |
| 274 | 61808 | M3_inf_ L | 0.35 |  |  | 0.35 |  | 112 | 0.711512 | 45 | -19.68 | 0.09 | 12.16 | 0.00 |
| 288 | 61999 | M2_sup_ L | 0.48 | 0.07 |  | 0.48 |  | 192 | 0.711810 | 93 | -18.74 | - | 12.44 | - |
| 288 | 62000 | M3_sup_ L | 0.06 |  |  | 0.06 |  | 126 | 0.711319 | 178 | -19.24 | - | 13.79 | - |
| 288 | 62014 | M2_inf_ R | 0.35 |  |  | 0.35 |  | 102 | 0.711246 | 148 | -18.97 | - | 14.23 | - |
| 702 | 70385 | M3_sup_ R | 0.16 |  |  | 0.16 |  | 88 | 0.712628 | 103 | -19.24 | - | 13.14 | - |
| 703 | 70388 | M3_sup_ L | 0.09 | - |  | 0.09 |  | 155 | 0.712222 | 98 | -19.48 | - | 11.67 | - |
| 706 | 70414 | M2 inf R | 0.43 |  |  | 0.43 |  | 162 | 0.711800 | 112.6 | -19.46 | 0.05 | 13.64 | 0.00 |
| 708 | 70421 | M2 sup L | 0.82 |  | 0.61 | 0.72 | 0.15 | 98 | 0.709574 | 46 | -19.43 | - | 11.50 | - |
| 713 | 70473 | M3 inf L | 0.55 |  | 0.48 | 0.52 | 0.05 | 107 | 0.709555 | 60 | -19.34 | - | 11.36 | 0.00 |
| 713 | 70551 | M2 inf R | 0.77 | - |  | 0.77 |  | 98 | 0.709978 | 31 | - | - | - | - |
| 720 | 70595 | M2_inf_ R | 0.36 |  |  | 0.36 |  | 113 | 0.710597 | 79 | -18.82 | 0.03 | 12.98 | 0.07 |
| 723 | 70600 | M3_inf_ R | 0.42 | - |  | 0.42 |  | 85 | 0.710541 | 38 | - | - | - | - |
| 728 | 70718 | M3? | 0.39 | 0.00 |  | 0.39 |  | 119 | 0.711641 | 68 | -19.19 | 0.02 | 13.11 | 0.07 |
| 729 | 70732 | M2_inf_ R | 0.47 |  |  | 0.47 |  | 79 | 0.710731 | 93 | -19.24 | 0.13 | 13.34 | 0.04 |
| 743 | 70826 | M3_sup_ L | 0.40 |  |  | 0.40 |  | 142 | 0.711632 | 77 | -18.66 | 0.08 | 13.88 | 0.00 |
| 745 | 70832 | M2_sup_ R | 0.35 |  |  | 0.35 |  | 146 | 0.711597 | 119 | -19.06 | 0.12 | 13.68 | 0.01 |
| 1001 | 80454 | M3_sup_ L | 0.43 | - |  | 0.43 |  | 87 | 0.711498 | 81 | - | - | - | - |
| 1002 | 80459 | M2_inf_ L | 0.65 |  |  | 0.65 |  | 102 | 0.709446 | 132 | -18.85 | 0.07 | 13.49 | 0.10 |
| 1008 | 80492 | M3_sup_R | 0.00 |  |  | 0.00 |  | 78 | 0.710402 | 82 | - | - | - | - |
| 1013 | 80510 | M2_sup_ L | 0.23 |  |  | 0.23 |  | - | 0.711240 | 136 | -19.68 | 0.03 | 13.01 | 0.06 |
| 1109 | 80822 | M2_inf_ dec | 0.54 |  |  | 0.54 |  | 67 | 0.711119 | 89.0 | -19.26 | 0.06 | 13.09 | 0.01 |
| 1120 | 80861 | M3_inf_ L | 0.22 |  |  | 0.22 |  | 105 | 0.709603 | 126 | -19.14 | 0.03 | 12.06 | - |
| 1134 | 80913 | M3_inf_ R | 1.04 |  |  | 1.04 |  | 83 | 0.709135 | 52 | -18.73 | 0.09 | 13.63 | 0.09 |
| 1137 | 80924 | M3_inf_ | 0.34 |  |  | 0.34 |  | 203 | 0.711831 | 124 | -19.37 | 0.01 | 11.97 | 0.03 |
| 1093 | 81059 | M2_sup_ L | 0.16 | - |  | 0.16 |  | 122 | 0.711626 | 109 | -18.86 | 0.06 | 14.01 | 0.12 |
| 1167 | 81112 | M3 | 0.28 |  |  | 0.28 |  | 112 | 0.711510 | 96 | -19.47 | 0.03 | 12.90 | 0.00 |
| 1174 | 81166 | M3_inf_ R | 0.34 |  |  | 0.34 |  | 135 | 0.712186 | 97 | -18.71 | - | 11.30 | - |
| 1192 | 81300 | M3_inf_ L | 0.50 | - |  | 0.50 |  | 113 | 0.710482 | 55 | -19.08 | 0.08 | 14.04 | 0.04 |
| 1000 | 81307 | M2_inf_ R | 0.34 | - |  | 0.34 |  | 129 | 0.711112 | 140 | - | - | - | - |
| 1202 | 81361 | M2_inf_ L | 0.33 | - |  | 0.33 |  | 99 | 0.711262 | 105 | -19.34 | 0.03 | 13.43 | 0.03 |
| 1204 | 81381 | M3_inf_ R | 0.55 | 0.04 |  | 0.55 |  | 102 | 0.712319 | 100 | -19.63 | 0.14 | 12.87 | 0.05 |
| 1209 | 81411 | M2_inf_ | 0.42 | - |  | 0.42 |  | 224 | 0.711334 | 122 | -19.61 |  | 13.81 | - |
| 1211 | 81419 | M3_inf_ L | 0.00 |  |  | 0.00 |  | 181 | 0.710110 | 198 | - | - | - | - |
| 1212 | 81428 | M2_inf_ L | 0.21 | 0.01 | 0.08 | 0.15 | 0.09 | 91 | 0.712782 | 119 | - | - | - | - |
| 1094 | 81633 | M2_inf_ R | 0.22 | 0.04 |  | 0.22 |  | 110 | 0.711290 | 157 | -18.66 | 0.04 | 14.27 | 0.05 |
| 1251 | 81702 | M2_inf_ R | 0.38 |  |  | 0.38 |  | 145 | 0.711406 | 109 | -19.14 | 0.07 | 14.17 | 0.04 |
| 1004 | 81732 | M2_sup_ L | 0.48 |  |  | 0.48 |  | 183 | 0.711685 | 115 | -18.63 | 0.02 | 15.92 | 0.04 |
| 1298 | 81935 | M3_inf_ L | 0.46 |  |  | 0.46 |  | 217 | 0.711179 | 117 | -19.47 | 0.09 | 12.76 | 0.10 |

Table S2: Isotope data for human teeth in Rennes, Brittany. Carbon and nitrogen isotope data come from a previous study (Colleter et al., in press). **δ^66^Zn_t_** : average Zn isotope values when all measurements are included. SD_A_= analytical standard error. SD_I_= standard error taking into account different sample preparation.

| Animal | **^87^Sr/^86^Sr** | | **δ^66^Zn_t_** | | **δ^13^C_t_** | | **δ^15^N_t_** | | **δ^34^S_t_** | |
| --- | --- | --- | --- | --- | --- | --- | --- | --- | --- | --- |
| pig | 0.711640 | | 0.65 | | -20.8 | | 12.7 | | NA | |
| cat | 0.711584 | | 0.66 | | -19.2 | | 12.4 | | 14.8 | |
| dog | 0.712700 | | 0.50 | | -19.6 | | 11.0 | | 13.3 | |
| veal | 0.713756 | | 0.93 | | -22.0 | | 8.6 | | 11.4 | |
| cow | 0.714669 | | 0.85 | | -21.7 | | 9.6 | | 12.1 | |
| sheep | 0.712420 | 1.09 | | -21.6 | | 8.0 | | 12.0 | |  |

Table S3: Isotope data for animal teeth in Rennes, Brittany. The animal teeth come from the refuse midden. Carbon, nitrogen and sulphur isotope data come from a previous study (Colleter et al., in press).

| **Standard** | **type** | **^87^Sr/^86^Sr measured** | **SD** | **n** | **^87^Sr/^86^Sr expected** | **SD** | **n** |
| --- | --- | --- | --- | --- | --- | --- | --- |
| **SRM 1486** | Bone meal | 0.709290 | 0.000019 | 5 | 0.709299 | 0.000027 | In house long term measurement on 94 samples |
|  |  | δ^66^Zn_measured_ | SD | n | δ^66^Zn_expected_ | SD | n |
| **AZE** | Dentine, in house | 1.51‰ | 0.14 | 5 | 1.50 ‰ | 0.04 | 1 |
| **SRM 1400** | bone ash | 1.0 | 0.04 | 3 | Never measured |  |  |

Table S4: Isotope data of standards (Zn and Sr isotope ratios). Zn isotope values are expressed relative to the standard JMC Lyon.
